# Halloysite-Assisted Delivery of Cannabidiol for the Management of Temporomandibular Pain: A Pilot Study

**DOI:** 10.3390/jcm15010145

**Published:** 2025-12-24

**Authors:** Karolina Walczyńska-Dragon, Aleksandra Grzyb, Karolina Dawiec, Maria Pawłowska, Jakub Fiegler-Rudol, Paweł Hadzik, Aleksandra Nitecka-Buchta, Stefan Baron

**Affiliations:** 1 Department of Temporomandibular Disorders, Medical University of Silesia in Katowice, 2 Traugutta Sq., 41-800 Zabrze, Poland; 2 Student Scientific Society, Department of Temporomandibular Disorders, Medical University of Silesia in Katowice, 41-800 Zabrze, Poland; 3 Department of Physiotherapy, Opole University of Technology, Prószkowska Street 76, 45-758 Opole, Poland

**Keywords:** CBD, cannabidiol, EMG, halloysite, TMD, bruxism, orofacial pain

## Abstract

**Background**: Bruxism and temporomandibular disorders (TMD) are commonly associated with increased masticatory muscle activity and pain. Cannabidiol (CBD) exhibits analgesic, myorelaxant, and anti-inflammatory properties, while halloysite may enhance mucosal delivery and bioavailability. **Methods**: In a randomized, double-blind pilot trial, 20 adults with TMD applied either a CBD gel or a CBD plus halloysite gel nightly for 6 weeks. Masseter muscle activity was recorded using surface electromyography (sEMG) at baseline and post-treatment. **Results**: Both formulations significantly reduced masseter sEMG activity. The mean decrease was 37.95% with CBD alone (SD = 9.37) and 37.41% with CBD plus halloysite (SD = 5.44). Minimum reductions were 20.44% and 20.02%, and maximum reductions reached 55.16% and 82.52%, respectively. Reductions were bilateral and comparable between right and left sides. Differences between formulations were not statistically significant by *t*-test (*t*(8) = 1.613, *p* = 0.145) or Mann–Whitney *U* test (*p* > 0.5). However, variability was lower with the CBD plus halloysite formulation, suggesting a more consistent response. A sex effect reached significance within one formulation (*t*(8) = 2.315, *p* = 0.049), while no sex difference was observed in the other. Treatment duration did not correlate with effect size for either gel (Spearman’s rₛ = 0.213 and −0.071, both *p* > 0.5). No adverse events were reported. **Conclusions**: Nightly intraoral CBD and CBD plus halloysite gels reduced masseter sEMG in adults with TMD, with similar mean efficacy and lower response variability for CBD plus halloysite. These pilot data support further adequately powered, placebo-controlled trials to confirm efficacy, define optimal dosing, and clarify subgroup effects. The trial registration number registered prospectively is NCT05562635 (accessed on 31 August 2022).

## 1. Introduction

### 1.1. Background and Rationale

Temporomandibular disorders (TMD) is a collective term for musculoskeletal conditions involving pain and dysfunction in the masticatory muscles, temporomandibular joints (TMJ) and related structures. Patients suffering from TMD typically experience chronic pain, and often have co-occurring headaches, tinnitus and other ear disfunctions, chronic fatigue syndrome, depression and sleep disturbances [1]. TMD is the most common oral and facial pain condition, affecting up to 15% of adults and 7% of adolescents [2]. Chronic pain is the main reason TMD patients seek treatment. TMD can be associated with worsening overall health, depression and other psychiatric disorders, and can affect a patient’s quality of life [3]. Bruxism may serve as an aggravating factor in TMD, while psychosocial stress contributes significantly to its onset and severity [4].

Bruxism is classified as sleep (SB) or awake (AB) bruxism. Sleep bruxism is a sleep-related movement disorder with rhythmic (phasic) or non-rhythmic (tonic) masticatory activity. Awake bruxism is classified as repetitive or sustained tooth contact during wakefulness and is a behavior that may confer risk for clinical consequences rather than a disorder itself [5,6]. Importantly, sleep bruxism may provide physiological benefits in selected conditions: recent polysomnographic evidence indicates that SB episodes can support airway patency and salivary neutralization, thereby exerting protective effects in gastroesophageal reflux disease and obstructive sleep apnea [7]. Clinically, nocturnal bruxism is suggested by the presence of wear facets, particularly on incisors and canines, and may present with morning headaches or a sensation of upper-quarter muscle tension. These manifestations are associated with metabolic alterations in the masseter and lateral pterygoid muscles during nocturnal parafunctional activity [8]. Myofascial pain typically involves trigger points, palpable, pressure-sensitive nodules formed in response to trauma, local ischemia, and impaired perfusion. Sustained contraction within these loci perpetuates a cycle of reduced oxygenation and nociceptive activation [9]. Given these pathophysiological features, any compound intended to reduce muscle hyperactivity should ideally reach tissues as close as possible to ischemic loci. This consideration has stimulated interest in improving intraoral mucosal delivery strategies for cannabidiol (CBD), with the aim of increasing local bioavailability and enhancing therapeutic effects.

Nanomaterials, including lipid, polymer, porous, and clay-based nanoparticles, are increasingly explored in biomedical applications for their ability to modulate drug stability, transport, and release kinetics. Among them, nanoclays, such as halloysite, bentonite, kaolinite, laponite, and montmorillonite, are distinguished by their high porosity, large specific surface area, biocompatibility, and capacity for sustained release [10]. Halloysite, a naturally occurring aluminosilicate typically forming nanotubes or nanoplatelets, has been extensively investigated as a carrier for controlled drug delivery. One of the three major global deposits is in Dunino, Poland. Its characteristic two-layer structure, comprising siloxane and aluminol surfaces, provides a high density of reactive hydroxyl groups on the aluminol layer, facilitating molecular interactions, surface functionalization, and efficient loading. The greater exposure of the aluminol surface in nanoplatelets compared with nanotubes may further enhance loading efficiency and modulate release characteristics, potentially improving mucosal absorption of incorporated compounds such as CBD [11].

Despite the growing interest in CBD formulations, currently available oral products, including oils, tinctures, capsules, sprays, and toothpastes, are limited by the inherently low bioavailability of CBD and by poor retention in the dynamic oral environment. Conventional preparations are susceptible to rapid salivary washout, enzymatic degradation, and oxidation. Thus, a clear gap exists in the development of mucoadhesive, controlled-release delivery systems capable of maintaining CBD in prolonged contact with the buccal mucosa immediately adjacent to hyperactive muscles. 

Halloysite is biocompatible and safe for living tissues, as confirmed by numerous studies [11]. It has shown wide applicability as a carrier for controlled drug release, in tissue engineering, stem cell isolation, and as a reinforcing filler in dental composites. Its versatility and biocompatibility make it a promising material for advanced therapeutic development [12]. CBD is a non-psychoactive cannabinoid derived from the *Cannabis sativa* plant [13]. Cannabinoids act mainly through CB1 and CB2 receptors [8]. Both receptor types and their ligands are present in the central and peripheral nervous systems, and CB receptor expression has been identified in dental pulp and periodontal tissues [13]. Cannabidiol functions as a CB1 receptor antagonist, producing analgesic, anti-inflammatory, antiepileptic, antiemetic, and anxiolytic effects [14,15]. Despite growing interest in CBD-based therapies for TMD and bruxism, current intraoral products show limited therapeutic efficiency because CBD has low mucosal permeability, short residence time, and rapid salivary washout [11,12]. These constraints result in inconsistent local exposure and variable clinical effects. This gap highlights the need for delivery systems capable of maintaining CBD in sustained contact with the buccal mucosa near hyperactive masticatory muscles. Halloysite was selected because its surface chemistry and high loading capacity can support controlled release and improve stability of incorporated compounds. Although mechanistic confirmation is beyond the scope of this pilot trial, halloysite’s characteristics make it a plausible strategy for increasing the uniformity of local CBD exposure [14,15].

Although numerous CBD-containing oral products exist, their therapeutic potential is limited by poor and variable bioavailability. Halloysite represents a promising approach to broaden the pharmacological efficacy of CBD by enhancing absorption during localized intraoral application [16,17,18].

### 1.2. Objectives

This pilot study is, to our knowledge, the first randomized, double-blind evaluation of intraoral mucosal CBD delivered with a halloysite carrier in patients with painful TMD. It addresses a key gap in cannabinoid therapeutics for orofacial pain by testing a locally applied, mucoadhesive gel that aims to maximize mucosal residence and reduce systemic exposure. Halloysite was selected as a biocompatible nanoclay with high surface area and favorable loading and release characteristics, offering a translational path for controlled CBD delivery on the oral mucosa. Methodologically, the study uses standardized sEMG of the masseter muscles as an objective biomarker of muscle hyperactivity, with bilateral recordings, templated electrode placement, and blinded allocation to reduce measurement and allocation bias. The work also explores signal consistency and subgroup effects that are rarely examined in this field, including variability of response with a nanoclay carrier and preliminary sex-specific differences. By combining a novel delivery platform with objective physiology-based endpoints, this study provides an early proof of concept for mucosal CBD-halloysite formulations as a conservative, local therapy for TMD-related muscle hyperactivity and pain.

## 2. Materials and Methods

### 2.1. Study Design and Participants

This randomized, double-blind clinical pilot study was conducted at the Department of Temporomandibular Disorders, Medical University of Silesia in Katowice, Poland. Twenty participants (10 women and 10 men), all students of the Medical University of Silesia aged 22–28 years, were enrolled after fulfilling the inclusion and exclusion criteria (Table 1). The mean age in Group 1 was 24.2 years, and in Group 2, 23.8 years (Table 2). Participants were allocated to one of two intervention arms using a simple allocation-by-draw procedure. Identical, opaque containers were prepared in advance and labeled with even or odd numerical codes corresponding to the two formulations. Containers were indistinguishable in appearance. Allocation occurred when each participant selected a sealed container. The allocation sequence was not computer-generated, and no block or stratified randomization was applied. Preparation and labeling of containers were performed prior to enrollment by personnel not involved in outcome assessment. Because of the simple draw-based method and small sample size, full allocation unpredictability cannot be guaranteed.

### 2.2. Inclusion and Exclusion Criteria

The inclusion and exclusion criteria are summarized in Table 1. Key inclusion requirements were: age > 18 years, general good health, and confirmed TMD muscle-related diagnosis according to the Polish version of the Research Diagnostic Criteria for Temporomandibular Disorders (RDC/TMD). Major exclusion factors included active lesions of the oral mucosa, hypersensitivity to study substances, chronic use of analgesics or muscle relaxants, smoking, and history of cannabis abuse.

### 2.3. Characteristics of the Study Group

The study included a total of 20 participants, divided equally into two groups. Each group consisted of 10 individuals, with an equal sex distribution of 5 women and 5 men. The mean age of participants in Group 1 was 24.2 years, while in Group 2 it was 23.8 years.

### 2.4. Study Protocol

The study consisted of two clinical visits conducted in a standardized, quiet environment, with all examinations performed in a seated position on a dental chair. During the baseline visit, participants underwent qualification according to inclusion and exclusion criteria, followed by a detailed medical history, extraoral and intraoral assessment, and functional evaluation of the stomatognathic system. Subsequently, superficial electromyography (sEMG; Neurobit Optima System) was used to record bilateral masseter muscle activity. Eligible participants were randomly allocated to one of two groups: Group 1, receiving a preparation containing CBD, or Group 2, receiving a preparation containing CBD combined with halloysite. Randomization was carried out by drawing containers indistinguishable in appearance, which were prefilled with either CBD gel or CBD with halloysite, labelled with an even or odd number corresponding to one of the intervention arms. The procedure was accompanied by detailed usage instructions. Proper storage guidelines were also provided to each participant.

To ensure safety, all participants performed an independent allergy test prior to intraoral use by applying a small amount of the preparation (approximately the size of a coffee bean) to the volar forearm for 24 h. In the absence of local reactions such as erythema, itching, or swelling, participants proceeded with intraoral application. Each subject was instructed to apply the preparation nightly to the buccal mucosa near the masseter muscle of both cheeks, covering an area of approximately 3 × 3 cm, for a period of 6 weeks. The formulations were dispensed in standardized containers (Uno Dose, Eprus PN-EN ISO 15378:2018 [19]), designed to ensure accurate dosing. One full rotation of the dispensing knob released 0.2 g of gel, as confirmed through laboratory calibration, corresponding to the recommended dose for one side per evening application.

To monitor adherence, participants were asked to complete daily logs documenting each application, allowing verification that the preparation was used consistently each evening.

The follow-up visit was scheduled after approximately 6 weeks and included repetition of the entire baseline protocol, comprising extraoral and intraoral examination as well as bilateral masseter muscle activity measurements using sEMG.

### 2.5. Electromyographic Recordings

Surface electromyography (sEMG) measurements were performed using a four-channel Neurobit Optima 4.0 Portable Physiological Data Acquisition System (Neurobit System, Gdynia, Poland) equipped with BioExplorer software (Version 1.7). For each participant, five disposable Ag/AgCl electrodes (25.7 mm, Sorimex, Toruń, Poland), constructed with polyethylene foam and stainless-steel snaps, were employed. Two electrodes were placed bilaterally along the course of the fibers of the right and left masseter muscles, with one located below the zygomatic arch and the other near the mandibular angle, maintaining an interelectrode distance of at least 10 mm. A reference electrode was positioned in the cervical region. Electrode placement was standardized using individualized paper templates prepared for each participant, based on anatomical landmarks palpated during clinical examination, ensuring reproducibility across sessions. Skin was prepared according to SENIAM guidelines, which included shaving, if necessary, followed by cleansing and disinfection prior to electrode application.

Recordings were obtained both at rest and during maximum voluntary clenching. Real-time data transmission to the computer was executed, and for each muscle the highest sEMG values from two consecutive measurements were used for analysis. Measurements were performed separately for the right and left masseter muscles. To minimize transcription and evaluation errors, all datasets were independently verified and double-checked by two researchers. Variability of results across sessions are summarized in Table 1.

### 2.6. Formulation of Study Preparations

The study preparations were developed under controlled laboratory conditions to ensure reproducibility and stability of the tested gels. The base matrix was Celugel (Actifarm, Permit No. 30050), selected for its robust mucoadhesive and bioadhesive properties, which provide prolonged contact with the oral mucosa and enable consistent therapeutic action. Celugel is a transparent hydrophilic substrate characterized by a pH of 4.5–6.0, an undetectable odor, and a lightweight formulation. Its composition includes hydroxyethylcellulose (HEC), glycerol, sorbic acid, potassium sorbate, and purified water. The recommended addition of active therapeutic substances to Celugel is typically within the range of 5–10% [12].

The final study gels were prepared in two variants. Both contained Celugel at 160 g (80%) and liquid paraffin at 20 g (10%). Liquid paraffin, incorporated as an emollient additive, enhances the texture of the gel and improves its spreading ability. It is odorless, colorless, and commonly used in emulsion systems for its safety profile and moisturizing properties [13]. The active therapeutic component was CBD isolate (Hempla Sp. z.o.o., Lublin, Poland), derived from *Cannabis sativa* L. The isolate was added at a concentration of 10 g (5%). In the second preparation, corresponding to Group 2, halloysite was introduced at 10 g (5%). Halloysite, a naturally occurring aluminosilicate clay, was incorporated to provide potential synergistic effects with CBD through its structural and adsorptive properties.

Thus, the final compositions of the study gels were as follows:

- Group 1 (CBD preparation): Celugel 160 g (80%), liquid paraffin 20 g (10%), CBD isolate 10 g (5%).
- Group 2 (CBD + halloysite preparation): Celugel 160 g (80%), liquid paraffin 20 g (10%), CBD isolate 10 g (5%), halloysite 10 g (5%).

Both formulations were prepared in compliance with pharmaceutical standards and delivered in calibrated dosing containers to ensure accuracy and reproducibility in clinical use.

### 2.7. Ethical Approval

The study protocol was approved by the Bioethical Committee of the Silesian Medical Chamber in Katowice, Poland (No. KB1/66/II/20/21) and was prospectively registered at ClinicalTrials.gov (NCT05562635) (accessed on 31 August 2022). The research was conducted in compliance with the Declaration of Helsinki and the International Conference on Harmonisation–Good Clinical Practice guidelines (ICH-GCP). All participants received verbal and written information and provided written informed consent prior to enrollment.

### 2.8. Assessment of Normality and Choice of Statistical Tests

Normality of data distributions was evaluated using the Shapiro–Wilk test. Variables meeting the assumption of normality and homogeneity of variance were analyzed with parametric methods, including independent-samples *t*-tests and Pearson’s correlation. For variables deviating from normality, non-parametric alternatives were applied: the Mann–Whitney *U* test for group comparisons and Spearman’s rank correlation for associations. This approach ensured that statistical procedures were aligned with the distributional properties of the data, while subgroup analyses with small sample sizes (*n* = 5 per gender) were explicitly treated as exploratory. Because the sample size was small, results of normality testing should be interpreted with caution, as Shapiro–Wilk has limited power with n close to 10. For this reason, non-parametric analyses were included for all key comparisons to complement parametric findings.

## 3. Results

Surface electromyography measurements showed a consistent reduction in masseter muscle activity after six weeks of treatment with both CBD and CBD–halloysite formulations. All participants demonstrated decreased sEMG values, and no increase in muscle activity was recorded in either group after treatment.

### 3.1. General Effect of Treatment

Both tested preparations produced a significant reduction in sEMG activity of the masseter muscles. The mean decrease in overall muscle activity was 37.95% (SD = 9.37) for the CBD group and 37.41% (SD = 5.44) for the CBD + halloysite group. The reductions were statistically comparable, indicating similar efficacy of both formulations in lowering muscle activity. The minimum recorded decrease was 20.44% in the CBD group and 20.02% in the CBD + halloysite group, while the maximum decrease reached 55.16% and 82.52%, respectively. Although mean values were nearly identical, the CBD–halloysite formulation produced a notably higher maximum reduction, suggesting greater responsiveness in selected individuals. No participant reported local irritation, mucosal lesions, or systemic adverse effects during or after the intervention period.

### 3.2. Gender-Specific Analysis

Both male and female participants exhibited substantial reductions in masseter sEMG activity. In the CBD group, the average decrease was 37.95% (SD = 5.58) among women and 36.75% (SD = 3.18) among men. In the CBD + halloysite group, the average decrease was 30.81% (SD = 3.91) for women and 44.01% (SD = 3.72) for men. However, the low standard deviations within both formulations confirm the consistency and reliability of the observed effects.

### 3.3. Side-Specific Changes (Right vs. Left Masseter)

Reductions in sEMG activity were observed bilaterally in all participants. In the CBD group, the average reduction was 40.69% for the right masseter and 37.74% for the left. In the CBD + halloysite group, the corresponding values were 37.61% and 37.20%. This symmetry indicates uniform absorption and distribution of both formulations across the oral mucosa.

### 3.4. Variability of Results

Analysis of standard deviations showed lower variability in the CBD + halloysite group (SD = 5.44) compared with the CBD-only group (SD = 9.37), suggesting that halloysite contributed to a more stable and uniform therapeutic effect. Similar trends were observed across gender subgroups, with smaller standard deviations in participants receiving the CBD–halloysite formulation. These findings may indicate improved mucosal retention and controlled release of CBD when combined with halloysite nanotubes.

In both groups, reductions in muscle activity were observed for the right and left sides. In the CBD group (Group 1), the overall minimum decrease was 20.44%, with an average reduction of 37.95% (40.69% for the right and 37.74% for the left masseter) and a maximum reduction of 55.16% (50.44% right, 68.8% left). Among women, the mean decrease was slightly higher at 39.15%, while for men it was 36.75%. In the CBD plus halloysite group (Group 2), the overall minimum decrease was 20.02%, and the mean reduction reached 37.41% (37.61% right, 37.20% left), with a maximum decrease of 82.52% (71.01% right, 94.02% left). When analyzed by gender, women showed an average reduction of 30.81%, whereas men demonstrated a greater response, with a mean decrease of 44.01%. In both groups, muscle activity declined symmetrically on both sides, and no cases of increased sEMG activity were recorded after treatment. As shown in Table 2, the CBD + halloysite group demonstrated lower standard deviations in average sEMG reduction compared with the CBD-only group, indicating a more consistent and uniform therapeutic response across participants, particularly among men.

### 3.5. Sex Differences Within the Halloysite Group

An independent samples *t*-test was conducted to compare the mean reduction in masseter sEMG activity between women and men treated with the halloysite-based preparation. The analysis revealed a statistically significant difference, *t*(8) = 2.315, *p* = 0.0493, with women (M = 44.26, SD = 5.56) showing a greater mean reduction in muscle activity than men (M = 37.12, SD = 4.08). These results suggest that sex influenced treatment response, with a stronger therapeutic effect observed in female participants.

### 3.6. Sex Differences Within the CBD Group

A non-parametric Mann–Whitney U for the CBD-only group gave *p* > 0.05 for all comparisons. Thus, no statistically significant difference was found between female and male participants for the CBD formulation.

### 3.7. Comparison Between Halloysite and CBD Formulations Comparison Between Halloysite and CBD Formulations by Gender (Female Subgroup)

An independent samples *t*-test was performed to compare the overall mean reduction in masseter sEMG activity between the two formulations. The results showed *t*(8) = 1.613, *p* = 0.145, with mean reductions of 44.26% (SD = 5.56) for the CBD + halloysite group and 34.67% (SD = 12.08) for the CBD-only group. Although the halloysite formulation demonstrated a higher average decrease, this difference was not statistically significant at α = 0.05. The variance ratio (F = 4.73, *p* = 0.161) confirmed homogeneity of variances. Overall, no significant difference in mean effectiveness was observed between the two preparations, and the small sample size (*n* = 5 per group) may have limited the statistical power to detect moderate effects.

### 3.8. Mann–Whitney U Tests (Non-Parametric Confirmation) Comparison Between Halloysite and CBD Formulations by Gender (Male Subgroup)

The comparison between the halloysite and CBD groups yielded U = 10, Z = 0.418, *p* = 0.676, indicating no significant difference. Similarly, the analysis of average percentage reduction produced U = 41, Z = 0.643, *p* = 0.521, also showing no significant difference between treatments. These results confirm that the median values did not differ significantly between the two formulations. The small effect size (Z ≈ 0.64) suggests that even with a larger sample, the difference would likely remain non-significant.

### 3.9. Relationship Between Duration of Use and Treatment Effect

Spearman’s rank correlation was used to examine the relationship between treatment duration (in days or months) and the average percentage reduction in masseter sEMG activity. The analysis showed *rₛ* = 0.213, *p* = 0.554 for the CBD + halloysite group, and *rₛ* = −0.071, *p* = 0.845 for the CBD-only group, indicating no significant correlations.

### 3.10. Summary of Tests

Table 3 summarizes the statistical comparisons carried out in this study.

## 4. Discussion

### 4.1. Results in the Context of Other Evidence

To the authors’ knowledge, this pilot study is the first randomized, double-blind investigation to examine an intraoral cannabidiol (CBD) formulation incorporating halloysite in patients with muscle-related temporomandibular disorders (TMD). The primary objective was to compare the effects of a CBD-only gel and a CBD plus halloysite gel on masseter muscle activity assessed by surface electromyography (sEMG). The findings demonstrate that nightly intraoral application of both formulations was associated with a reduction in masseter muscle activity over the six-week intervention period. Mean reductions were similar between groups, amounting to 37.95% in the CBD-only group and 37.41% in the CBD plus halloysite group. Decreases were observed bilaterally, with comparable reductions on the right and left sides in both groups, indicating a consistent local effect across masseter muscles. No participant exhibited increased muscle activity after treatment, and no local or systemic adverse effects were reported, supporting the short-term tolerability of both preparations for intraoral use. The magnitude of reduction observed in the CBD-only group is consistent with earlier reports demonstrating decreases in masseter sEMG activity following topical CBD application. In particular, a prior randomized clinical trial reported a mean reduction of approximately 42.1%, which closely aligns with the present findings [20]. Together, these results reinforce existing evidence that locally applied CBD may reduce masticatory muscle hyperactivity associated with TMD. Although mean effects were comparable between formulations, the CBD plus halloysite group showed lower dispersion of individual responses and a wider range of observed reductions. Given the very small sample size, these features are reported descriptively and should not be interpreted as evidence of superior efficacy or as proof of a mechanistic advantage. While halloysite has been described in the material science literature as a nanoclay with properties that could support sustained release or altered mucosal interaction, no pharmacokinetic, release-profile, or tissue concentration data were collected in this study. Therefore, any suggestion that halloysite enhanced CBD bioavailability, mucosal retention, or stability remains hypothetical and cannot be inferred from the present data. Exploratory sex-specific analyses indicated a statistically significant difference between women and men within the CBD plus halloysite group, whereas no sex-related differences were observed in the CBD-only group. Given that each subgroup consisted of only five participants, these findings must be interpreted with considerable caution and should be regarded as hypothesis-generating rather than confirmatory. Biological explanations, such as sex-related differences in cannabinoid receptor expression or mucosal permeability, remain speculative and require targeted investigation in adequately powered studies. No significant association was observed between treatment duration and the magnitude of sEMG reduction in either group, suggesting that the observed effects may develop within the initial weeks of application. However, the narrow range of treatment duration and the limited sample size preclude firm conclusions regarding time-dependence. Overall, the present pilot data indicate that both CBD and CBD plus halloysite intraoral gels are associated with reductions in masseter muscle activity in young adults with TMD. While the addition of halloysite was associated with lower variability in observed responses, this finding should be interpreted descriptively and not as evidence of a mechanistic or clinical advantage. The small, homogeneous sample and the absence of a placebo control limit generalizability. Larger, placebo-controlled trials incorporating pharmacokinetic or release assessments, broader age ranges, and stratified analyses by sex and baseline muscle activity are required to determine efficacy, mechanism, and clinical relevance [9,21]. Within these constraints, the study provides preliminary evidence supporting further investigation of localized cannabinoid-based approaches for muscle-related orofacial pain conditions such as TMD and bruxism [20,21,22,23].

### 4.2. Limitations of the Study

This pilot study has several important limitations. The sample was small, homogeneous, and limited to healthy university students aged 22 to 28, which restricts external validity. The study duration was short and adherence was self-reported, so durability of effect and true exposure are uncertain. There was no placebo arm and no stratified randomization, which increases the risk of bias and chance imbalances between groups. Although electrode placement was standardized, sEMG is sensitive to positioning and skin impedance, so residual measurement error is possible. Only one CBD dose and a single halloysite concentration were tested, with no pharmacokinetic or release profile assessment, so dose response and mechanism cannot be inferred. Finally, potential confounders such as stress, sleep, caffeine, and parafunctional habits were not systematically controlled, and safety conclusions are limited by the small sample and brief follow up. Self-reported logs may overestimate adherence, especially in studies without electronic monitoring, which should be considered when interpreting effect magnitude and variability. Randomization was implemented using a simple container-drawing procedure rather than a computer-generated sequence, and allocation concealment did not meet CONSORT standards; therefore, residual allocation predictability cannot be excluded.

## 5. Conclusions

Both CBD and CBD-halloysite preparations, when applied to the masseter muscle, have been shown to reduce its surface electromyography (sEMG) activity, indicating a decrease in muscle tension. This effect suggests that these preparations may hold therapeutic potential for managing hyperactivity and tension in the masticatory muscles of patients with temporomandibular disorders (TMD). Given the chronic nature and often complex etiology of TMD, incorporating CBD-based treatments could provide an alternative or complementary approach to traditional therapies, potentially benefiting patients with muscle-related pain and dysfunction. However, additional research is crucial to determine the optimal formulation, dosage, and frequency of CBD and CBD-halloysite application. Long-term studies are especially needed to assess the safety and efficacy of these compounds over extended use. Future investigations should also consider individual patient responses and the influence of factors such as age, gender, metabolic rate, and any concurrent health conditions that could impact treatment outcomes.

## Figures and Tables

**Table 1:** The inclusion and exclusion criteria.

Inclusion Criteria	Exclusion Criteria
1. Patient’s agreement to participate in the study	1. Presence of wounds on the buccal mucosa
2. Age over eighteen years	2. Allergy to cannabis preparation, paraffin, or other study substances
3. General good health	3. Marijuana addiction
4. Positive diagnosis of temporomandibular disorder (Polish RDC/TMD)-muscle related	4. Tobacco smokers
	5. Long-term use of analgesics or drugs affecting muscle function
	6. Use of medications which interact with CBD

**Table 2:** Standard deviations for average decrease in masseter sEMG activity by preparation type and gender.

Variance (SD^2^)	Standard Deviation (SD)	Group	Preparation
29.55	5.44	General	CBD + Halloysite
15.27	3.91	Women	CBD + Halloysite
13.85	3.72	Men	CBD + Halloysite
87.79	9.37	General	CBD
31.09	5.58	Women	CBD
10.14	3.18	Men	CBD

**Table 3:** Summary of the statistical analysis used.

Comparison	Test	Statistic	df/*N*	*p*-Value	Result
Halloysite: women vs. men	*t*-test	*t* = 2.315	8	0.049	Significant
CBD: women vs. men	Mann–Whitney	*U* = 9	*N* = 10	0.530	Not significant
Halloysite vs. CBD (women)	*t*-test	*t* = 1.613	8	0.145	Not significant
Halloysite vs. CBD (men)	Mann–Whitney	*U* = 10	*N* = 10	0.676	Not significant
Average % decrease (Halo vs. CBD)	Mann–Whitney	*U* = 41	*N* = 10	0.521	Not significant
Duration vs. reduction (Halloysite)	Spearman	rₛ = 0.213	*N* = 10	0.554	Not significant
Duration vs. reduction (CBD)	Spearman	rₛ = −0.071	*N* = 10	0.845	Not significant

## Data Availability

The raw data supporting the conclusions in this article will be made available by the authors on request.
